# Investigating the Effect of One Year of Learning to Play a Musical Instrument on Speech-in-Noise Perception and Phonological Short-Term Memory in 5-to-7-Year-Old Children

**DOI:** 10.3389/fpsyg.2019.02865

**Published:** 2020-01-10

**Authors:** Douglas MacCutcheon, Christian Füllgrabe, Renata Eccles, Jeannie van der Linde, Clorinda Panebianco, Robert Ljung

**Affiliations:** ^1^Department of Building, Energy and Environmental Engineering, Högskolan i Gävle, Gävle, Sweden; ^2^Department of Music, University of Pretoria, Pretoria, South Africa; ^3^School of Sport, Exercise and Health Sciences, Loughborough University, Loughborough, United Kingdom; ^4^Department of Speech-Language Pathology and Audiology, University of Pretoria, Pretoria, South Africa

**Keywords:** speech in noise, phonological short-term memory, musical training, children, cognition

## Abstract

The benefits in speech-in-noise perception, language and cognition brought about by extensive musical training in adults and children have been demonstrated in a number of cross-sectional studies. Therefore, this study aimed to investigate whether one year of school-delivered musical training, consisting of individual and group instrumental classes, was capable of producing advantages for speech-in-noise perception and phonological short-term memory in children tested in a simulated classroom environment. Forty-one children aged 5–7 years at the first measurement point participated in the study and either went to a music-focused or a sport-focused private school with an otherwise equivalent school curriculum. The children’s ability to detect number and color words in noise was measured under a number of conditions including different masker types (speech-shaped noise, single-talker background) and under varying spatial combinations of target and masker (spatially collocated, spatially separated). Additionally, a cognitive factor essential to speech perception, namely phonological short-term memory, was assessed. Findings were unable to confirm that musical training of the frequency and duration administered was associated with a musicians’ advantage for either speech in noise, under any of the masker or spatial conditions tested, or phonological short-term memory.

## Introduction

Children receive their education in acoustic environments in which background noise is nearly always present. Classroom noise is known to cause distraction and annoyance in children, but its primary effect is a reduction in speech intelligibility (for reviews, see Shield and Dockrell, 2003; Klatte et al., 2013), with a consequently negative impact on academic achievement (Shield and Dockrell, 2008). In typically developing children, the ability to cope with speech in noise (SiN) has been linked to individual differences in cognitive and language abilities (Nelson et al., 2005; Strait et al., 2012; MacCutcheon et al., 2019), age (Corbin et al., 2016), gender (Prodi et al., 2019), and supra-threshold auditory processing abilities (Lorenzi et al., 2000), as well as environmental factors, including reverberation and the spatial, spectral and temporal characteristics of the background noise (MacCutcheon et al., 2018, 2019; McCreery et al., 2019).

Many studies have focused on how manipulating the acoustic environment can improve children’s attention to verbal instructions, self-rated ability to cope with noise, speech reception thresholds (SRTs) and cognitive performance (DiSarno et al., 2002; Purdy et al., 2009; Dockrell and Shield, 2012; Prodi et al., 2019). Contrastingly, the aim of the present study is to investigate whether musical training can improve individual characteristics of the listener that contribute to speech perception (e.g., auditory, linguistic and cognitive abilities) and thereby mitigate speech-intelligibility challenges posed by noise.

Musical training has been suggested as a possible candidate for improving auditory, linguistic and cognitive abilities (Patel, 2011; Tallal, 2014) because a multitude of studies indicate that adults and children with musical training show greater motor, cognitive, linguistic and auditory skills (for a review, see Benz et al., 2016), referred to as the “musicians’ advantage” (Bas̨kent and Gaudrain, 2016; Talamini et al., 2017). Indeed, a musicians’ advantage for SiN perception has been reported by a number of studies in adults and children (Parbery-Clark et al., 2009; Strait et al., 2012, 2013; Bidelman et al., 2014; Kraus et al., 2014; Slater et al., 2015; Bas̨kent and Gaudrain, 2016). However, there are also a substantial number of studies that failed to find strong evidence in favor of advantages in musicians (Strait et al., 2012; Fuller et al., 2014; Ruggles et al., 2014; Boebinger et al., 2015; Fleming et al., 2019; Zendel et al., 2019).

Despite diverging findings, there is a compelling theoretical basis for the possibility that musical training could improve speech perception. Indeed, due to the similarity of the acoustic features of music and speech, these stimuli are processed by the same brain networks (Patel, 2011). For example, both music and speech perception require the processing of fluctuations in the amplitude envelope of the acoustic signal (Patel, 2011) to discriminate musical notes and phrases and segments of syllables and words, respectively. Additionally, pitch processing (the ability to perceptually discriminate between frequencies) is both an essential aspect of the emotional and linguistic content of speech as well as the harmonic and melodic content of music.

How and why abilities developed through musical training might lead to improvements in SiN processing is currently still unknown. In this study, we consider three possibilities. The first is that musical training confers benefits for dealing with energetic and/or informational maskers; the second is that musical training improves spatial listening; and the third is that musical training confers benefits for SiN perception by improving mediating cognitive processes.

Noise presents a challenge for speech perception as a consequence of the acoustic and spatial characteristics of the masker. Energetic maskers reduce speech intelligibility, while informational maskers reduce speech perception due to acoustic similarity with the target speech, resulting in perceptual confusion (Brungart, 2001; Wightman and Kistler, 2005; Wightman et al., 2006; MacCutcheon et al., 2019), and informational interference (Dole et al., 2012; Stone et al., 2012). Meanwhile, localization cues provided by the spatial separation of the target speech from the masker can improve intelligibility because timing and level differences between the two ears assist with sound segregation (Litovsky, 2005; Johnstone and Litovsky, 2006); referred to as “spatial release from masking” (Freyman et al., 1999; Hawley et al., 2004). However, assessments of the potential for musical training to help speech perception under these acoustic and spatial conditions have produced mixed results (Parbery-Clark et al., 2009; Strait et al., 2012; Swaminathan et al., 2015) and there is a dearth of longitudinal studies in children in the literature.

The development of SiN perception occurs in conjunction with cognitive development (Hall et al., 2002; Bradley and Sato, 2008; Neuman et al., 2010). According to the Ease of Language Understanding model (Rönnberg et al., 2008), noise places demands on cognitive processing of speech as working memory resources are required for assisting with the matching of incoming phonological information with phonological representations stored in long term memory. Meanwhile, explicit processing resources are also used for making guesses (informed by prior knowledge and experience as well as contextual factors) that might provide clues as to the nature of the missing input. This turns a relatively automatic task into a cognitively demanding, effortful task. Both cross-sectional and longitudinal studies have shown musical-training-induced improvements in cognitive functioning in adults and children (Benz et al., 2016). In particular, phonological short-term memory processes essential for SiN perception seem to be higher in child and adult musicians than in non-musician controls (Chan et al., 1998; Lee et al., 2007; Franklin et al., 2008; Strait et al., 2012, 2013; Bergman Nutley et al., 2014; Roden et al., 2014).

The present study builds longitudinally on a previous cross-sectional study by MacCutcheon et al. (2019). The study investigated whether individual differences in linguistic and cognitive abilities contribute to SiN perception in a variety of listening conditions, composed of different masker types and spatial configurations of the target speech and masker. Participants were typically developing children in early stages of development that are critical to the co-development of language (Rhyner, 2009) and speech perception (Johnstone and Litovsky, 2006). The results of MacCutcheon et al. (2019) indicated that, under certain listening conditions, memory span and expressive language provided benefits for SiN perception. The present study adds to these findings by longitudinally assessing the effect of 1 year of musical training on SiN perception and phonological short-term memory. Children attended one of two schools with equivalent academic curriculums, except that one school offered additional music lessons as part of the school curriculum while the other school offered additional sports activities. Based on the published literature, it was hypothesized that musical training minimizes the effect of energetic and/or informational masking on speech perception and maximizes the use of spatial cues, resulting in improved speech perception relative to the control group. An additional hypothesis was that musical training improves speech perception via improvements in phonological short-term memory.

Previous studies reporting evidence for a musicians’ advantage provided a higher frequency and longer duration of musical training for their participants than the present study. For example, Kraus et al. (2014)’s and Slater et al. (2015)’s children received up to 4 h of musical training per week for up to 2 years before a musicians’ advantage was discernible. Although lesson frequencies and lengths for beginners learning an instrument are by no means standardized, norms suggest that children who show an interest in music will initially receive a lesson in their primary instrument once per week. Beginner instrumental lesson times for young children are generally 30–60 min depending on the child’s innate musical abilities and attentional capacity as well as practicalities such as parental preferences and resources. As this range is more representative of what the majority of children engaging in musical activities at that age receive under “normal” circumstances, the present study hoped to ascertain a musicians’ advantage within a shorter timeframe and with a lower intensity of musical training than previous studies.

## Materials and Methods

### Participants

A total of 41 typically developing male school children participated in the study. On average, they were aged 6.3 years (standard deviation = 0.5 years, range: 5–7 years) at the start of the study, and had no history of cognitive, sensory or behavioral deficits, according to parental report. Parents of children in the participating schools received an information letter through the schoolteacher and agreed for their children to participate by providing written consent. Ethical approval for the study was granted by the University of Pretoria Research Ethics Committee, Approval 25071999 (GW20171130HS).

Prior to participation, all children were screened for hearing deficits. Normal hearing function was established using the smartphone hearing-screening application hearScreen^TM^ that detects hearing losses in excess of 20 dB Hearing Level at 1, 2, and 4 kHz with 97.8% reliability compared to standard manual audiometric procedures (Swanepoel et al., 2014). The application was run on Samsung Galaxy J2 mobile phones connected to Sennheiser HD280 Pro headphones.

### Musical Training and Control Groups

Twenty-six participants attended a music-focused school (the musical-training group) where they received up to 1 h per week of instrumental training over the course of a 38-week school year. The training was delivered by a qualified music teacher who used a combination of Kodaly and Orff methodologies.^1^ All children attended a 30-min group recorder lesson, and twelve (29%) children received a further 30-min individual piano or violin lesson. The remaining fifteen participants attended a sports-focused school (the control group) where they participated in extra-curricular sports (e.g., football, cricket, hockey and swimming) for 2–5 h per week. Both schools otherwise followed an equivalent Independent Examinations Board academic curriculum. As part of this curriculum, all children attended a weekly 30-min general group music lesson that did not involve instrumental training. None of the participants received additional musical training outside school.

The musical-training and control groups did not differ in age [*t*(39) = 1.38, *p* = 0.177, two-tailed], and socio-economic status as measured by maternal education level [*t*(39) = 0.39, *p* = 0.695, two-tailed]. Both groups were tested on the SiN and FDS tasks twice: once at the first assessment point (T1) when none of the participants had received any formal musical training, and then again at the second assessment point (T2) after attending their respective schools for 1 year. Between-group differences in language ability were also measured using the Renfew Action Picture Test (RAPT; Renfrew, 1980). This test consists of 10 pictures that must be verbally described (e.g., a girl hugging a teddy-bear), and the information and grammar content of the responses are scored out of 40 and 35 points, respectively. No group differences in language ability were detected at T1 [*t*(39) = −0.10, *p* = 0.922, two-tailed].

### Design

A 2 Groups (musical training vs. control) × 2 Assessment points (T1 vs. T2) × 2 Masker types [speech-shaped noise (SSN) vs. single talker] × 2 Spatial locations (collocated vs. spatially separated) mixed design was used. Speech-in-noise intelligibility was analyzed separately for each group at the two assessment points in each of the four listening conditions obtained by combining masker type and spatial location, as well as averaged across listening conditions.

### Tasks

#### Speech-in-Noise Perception

The SiN test was run on a DELL Latitude E6430 laptop, and the auditory stimuli were presented to the participants through a Focusrite Scarlett 2i2 audio interface and Sennheiser HD 650 headphones. All stimuli were pre-recorded and acoustics were simulated in a virtual classroom with a mean mid-frequency reverberation time *T*_30_ of 0.6 s using the software Room Acoustics for Virtual Environments (RAVEN; Schröder, 2011). Binaural room impulse responses were simulated based on a head-related transfer function measured from a child dummy head so that the virtually simulated environment was appropriate for the sample under investigation (Fels et al., 2004). Further details about the masker and the simulation of the virtual acoustic environment are reported in MacCutcheon et al. (2019). Speech identification was assessed using an adaptation of the “Children’s Coordinate Response Measure” software described in Vickers et al. (2016). The task was to identify two target words in the carrier sentence “show the dog where the [number word] [color word] is,” spoken by an adult male with an English accent. The color word was one of six colors (black, red, green, white, blue or pink) and the number word was a number between one and nine, with the exception of the disyllabic number seven. The location of the target talker was simulated to be at 0° azimuth. The target speech was accompanied by either a single male adult talker reading fictitious news items, or SSN with the same long-term average speech spectrum as the masking talker. The masker started and ended with the target sentence. Within the simulated virtual environment, each masker was either collocated with the target talker, or spatially separated to the right of the target talker, at +90° azimuth. SRTs for identifying the two target words correctly 50% of the time were assessed. The presentation level of the masker was fixed at 55 dB(A) while the presentation level of the target speech, initially set to 68 dB(A), was adaptively varied, using a 1-up, 1-down procedure (Levitt, 1971). Until the first incorrect response, the presentation level for the target speech was decreased by 8 dB. Then, a step size of 4 dB was used until the second incorrect response occurred. Thereafter, the step remained fixed to 2 dB. Each threshold run was composed of 48 sentences, corresponding to all possible color-number combinations. The SRT was computed as the mean of the final four reversals for a given threshold run.

#### Phonological Short-Term Memory Capacity

The “Number Repetition – Forward” subtest from the Clinical Evaluation of Language Fundamentals (CELF-4; Semel et al., 2003) was used to assess phonological short-term memory capacity. This version of a forward digit span (FDS) test required the participant to recall number sequences of varying length (from two to nine digits) in serial order. Initially, the sequence was composed of two digits and the sequence length was increased by one digit after two sequences of the same length were presented. The test was terminated once the participant incorrectly recalled two sequences of the same sequence length in a row, or completed all the lists. Each correctly recalled sequence was awarded a point, resulting in a maximum score of 16 points. Raw scores were converted to age-normed standard scores provided in the CELF-4 manual and all further analyses were conducted using standard scores.

### Experimental Procedure

Testing was conducted in a sound-isolated music room of one of the participating schools in the presence of an experimenter. For the SiN test, the graphical user interface showed a photograph of a dog beside six colored panels, each subdivided into nine numbered buttons representing all possible number and color combinations. Given their young age, participants were asked to repeat verbally the number and color they had heard, and the experimenter entered the responses for them by clicking the appropriate buttons on the user interface. The order of the four listening conditions was counterbalanced using a Latin square design. The FDS test was administered according to the protocol provided in the manual of the CELF-4.

## Results

Results for the two groups on the short-term memory task and the speech-perception task in the four different listening conditions and on average are given in Table 1 for the first and second assessment point.

**TABLE 1:** Group summary statistics in terms of mean, standard deviation (SD), and the lower and upper range of the 95% confidence interval (CI 95%) for performance on the forward digit span (FDS) test and speech-in-noise perception (SiN) test in each listening condition and on average.

**Test**	**Assessment point**	**Control group**			**Musical training group**		
		**Mean (dB)**	***SD***	**CI 95%**	**Mean (dB)**	***SD***	**CI 95%**
				**[lower, upper]**			**[lower, upper]**
FDS	T1	6.4	2.1	[5.2, 7.6]	7.9	2.0	[7.0, 8.7]
	T2	7.1	1.9	[6.1, 8.2]	8.2	1.5	[7.6, 8.8]
SiN: Collocated ^∗^ SSN	T1	−3.1	3.9	[−5.3, −0.9]	−3.1	4.7	[−5.0, −1.2]
	T2	−4.1	6.2	[−7.6, −0.7]	−5.6	2.8	[−6.7, −4.4]
SiN: Separated ^∗^ SSN	T1	−4.7	4.1	[−7.0, −2.4]	−3.8	5.3	[−5.9, −1.6]
	T2	−5.5	3.3	[−7.3, −3.7]	−7.1	2.5	[−8.1, −6.1]
SiN: Collocated ^∗^ single talker	T1	4.6	3.7	[2.5, 6.6]	5.2	3.6	[3.8, 6.7]
	T2	1.8	2.4	[0.5, 3.1]	1.8	3.8	[0.3, 3.4]
SiN: Separated ^∗^ single talker	T1	0.9	5.0	[−1.8, 3.7]	−0.7	5.5	[−3.0, 1.5]
	T2	−4.4	4.0	[−6.7, −2.2]	−4.4	3.6	[−5.9, −3.0]
SiN: Average	T1	−0.6	2.5	[−2.0, 0.8]	−0.6	3.3	[−1.9, 0.8]
	T2	−3.1	2.5	[−4.5, −1.7]	−3.8	1.9	[−4.6, −3.1]

### Baseline Performance

At the start of the study (i.e., at T1), the two groups did not differ significantly in SRTs averaged across the four listening conditions [*t*(39) = 0.017, *p* = 0.987, two-tailed]. However, there was a significant group difference on the FDS task [*t*(39) = −2.49, *p* = 0.013, two-tailed].

### Effect of Musical Training, Noise-Type, Spatial Factors and Time on Speech-in-Noise Perception

To determine whether additional musical training over 1 year yielded improvements in SiN perception, a repeated-measures analysis of variance (ANOVA) was conducted on the SRTs, with Group as the between-subjects factor, and Assessment point, Masker type and Spatial location as within-subjects factors. Estimated marginal means for all main effects and interactions are provided in Table 2.

**TABLE 2:** Estimated marginal mean speech-reception thresholds (SRTs), standard error (SE) and the lower and upper range of the 95% confidence intervals (CI 95%) for the main effects and interactions.

**Factors**	**Mean (dB)**	***SE***	**CI 95%**
			**[lower, upper]**
T1	−0.6	0.5	[−1.6, 0.4]
T2	−3.5	0.4	[−4.2, −2.7]
SSN	−4.6	0.4	[−5.5, −3.8]
Single talker	0.6	0.4	[−0.2, 1.4]
Collocated	−0.3	0.4	[−1.1, 0.5]
Separated	−3.7	0.5	[−4.6, −2.8]
Collocated ^∗^ SSN	−4.0	0.6	[−5.1, −2.8]
Collocated ^∗^ Single talker	3.4	0.5	[2.4, 4.3]
Separated ^∗^ SSN	−5.3	0.5	[−6.3, −4.3]
Separated ^∗^ Single talker	−2.2	0.6	[−3.4, −0.9]
SSN ^∗^ T1	−3.7	0.6	[−4.9, −2.4]
SSN ^∗^ T2	−5.6	0.4	[−6.5, −4.7]
Single talker ^∗^ T1	2.5	0.5	[1.4, 3.6]
Single talker ^∗^ T2	−1.3	0.4	[−2.2, −0.4]

The main effect of Assessment point indicated that both groups’ SiN perception was significantly better by 2.9 dB after 1 year [*F*(1,39) = 33.54, *p* < 0.001, $\eta_{p}^{2}$ = 0.46] consistent with findings that SiN perception improves with age (Hall et al., 2002). The significant main effect of Masker type [*F*(1,39) = 123.68, *p* < 0.001, $\eta_{p}^{2}$ = 0.76] indicated that the presence of a single talker led to an increase in SRTs by 5.2 dB compared to spectrally matched noise, across both groups and assessment points. The relative increase in perceptual difficulty experienced when the masker was a single talker is attributable to the acoustic similarity of the target and the masker with resulting informational interference (Dole et al., 2012; Stone et al., 2012), as well as the audible semantic content of the masker, which effectively captures attention in children (Cowan et al., 1999). The significant main effect of Spatial location [*F*(1,39) = 59.25, *p* < 0.001, $\eta_{p}^{2}$ = 0.60] indicated that across Group, Assessment point and Masker type factors, the average SRT in the collocated listening conditions was 3.4-dB higher compared to spatially separated listening conditions. This corroborates studies with adults and children indicating a benefit of spatially separating target and maskers (Litovsky, 2005; Johnstone and Litovsky, 2006).

The interaction between Assessment point and Group was not significant [*F*(1,39) = 0.59, *p* = 0.448, $\eta_{p}^{2}$ = 0.018], suggesting that the two groups did not differ in SiN perception, neither at baseline nor after providing additional musical training to one of the groups.

An interaction between Masker type and Spatial location and subsequent simple-effects analysis indicated that when the masker was SSN, speech in the collocated condition was significantly harder to perceive by 1.3 dB than in the spatially separated condition. When the masker was a single talker, this difference increased to 5.3 dB. This 4-dB difference in spatial release from masking shows that spatial cues are more helpful for children’s speech perception when dealing with realistic changing-state maskers that would often be present in the classroom environment. Furthermore, SRTs for the collocated condition were 7.3 dB higher in the presence of a single talker than in SSN, indicative of the burden that masker-target similarity and attention capture place on auditory stream segregation in children.

A significant interaction was found between Masker type and Spatial location [*F*(1,39) = 15.38, *p* < 0.001, $\eta_{p}^{2}$ = 0.28]. A simple-effect analysis revealed that spatially separating the masker from the target resulted in better SiN perception regardless of the type of masker: when the masker was SSN, speech in spatially separated conditions was significantly easier to perceive by 1.3 dB than when collocated [*F*(1,39) = 4.12, *p* = 0.05, $\eta_{p}^{2}$ = 0.095], but when the masker was a single talker, this increase between separated and collocated conditions grew to 5.5 dB [*F*(1,39) = 54.61, *p* < 0.001, $\eta_{p}^{2}$ = 0.5]. Furthermore, under both spatial conditions, speech masked by SSN was more intelligible than when masked by the single talker: when the masker was spatially separated, speech perception masked by SSN was 7 dB easier to discern than the single talker [*F*(1,39) = 21.39, *p* < 0.001, $\eta_{p}^{2}$ = 0.35], but this difference decreased to 3 dB when the masker was collocated but remained significant [*F*(1,39) = 94.91, *p* < 0.001, $\eta_{p}^{2}$ = 0.71].

Another significant interaction was found between Masker type and Assessment point [*F*(1,39) = 7.79, *p* = 0.008, $\eta_{p}^{2}$ = 0.17]. The simple effects analysis indicated that at both assessment points, SSN was the less challenging masker: SRTs at T1 were 6.2 dB better for SSN than for the single talker masker [*F*(1,39) = 102.02, *p* < 0.001, $\eta_{p}^{2}$ = 0.72], and at T2, the difference was reduced to 4.3 dB but remained significant [*F*(1,39) = 62.43, *p* < 0.001, $\eta_{p}^{2}$ = 0.62]. Furthermore, the improvement between the two assessment points was greater for the single talker than SSN: when the masker was SSN, the significant increase from T1 to T2 was almost 2 dB [*F*(1,39) = 9.04, *p* = 0.005, $\eta_{p}^{2}$ = 0.19], and this increase between assessment points grew to 3.8 dB when the masker was a single talker [*F*(1,39) = 47.41, *p* < 0.001, $\eta_{p}^{2}$ = 0.55]. This suggests that there are different developmental trajectories for coping with energetic and informational maskers. While the effect of the energetic masker (SSN) takes place in the auditory periphery, the effect of the informational masker (single talker) is located more centrally and probably involves cognitive processes. That the developmental effect was larger in the single-talker masker indicates that cognitive abilities which assist with SiN perception develop faster than those attributable to peripheral auditory processing.

### Effect of Musical Training on Phonological Short-Term Memory

A repeated-measures ANOVA, with the between-subjects factor Group and the within-subjects factor Assessment point, was conducted on the FDS scores to determine whether additional musical training yielded improvements in phonological short-term memory. There was a significant effect of Group [*F*(1,39) = 9.54, *p* = 0.004, $\eta_{p}^{2}$ = 0.197], with higher FDS score in the musical training group at both baseline and T2 [*t*(39) = −1.84, *p* = 0.022, two-tailed]. Within-subject effects indicated that, relative to T1, the average FDS score increased from 10.2 (SD = 3.1) to 10.4 points (SD = 2.5) at T2, but this increase was not significant [*F*(1,39) = 0.17, *p* = 0.684, $\eta_{p}^{2}$ = 0.004]. The interaction between Assessment point and Group was also not significant [*F*(1,39) = 0.41, *p* = 0.528, $\eta_{p}^{2}$ = 0.01]. Therefore, neither age-related development nor musical training produced improvements in FDS score in relation to baseline performance.

### Correlations Between Speech-in-Noise Perception and Phonological Short-Term Memory

The relationship between FDS scores and SRTs at T1 and T2 was assessed using two-tailed Pearson correlations. Results indicated significant covariance in only one of the listening conditions, namely when the SSN was collocated with the target speech at both T1 (*r* = −0.35, *p* = 0.026) and T2 (*r* = −0.45, *p* = 0.003). Correlations between FDS scores and SRTs in the other three conditions were non-significant (all *p* > 0.07).

## Discussion

### Effect of Musical Training on Speech-in-Noise Perception

The primary aim of this study was to assess whether additional weekly musical instrument training provided over the course of 1 year improves speech perception under the sorts of challenging acoustic conditions children could realistically expect to experience in a classroom. Namely, environments in which energetic and informational maskers in various spatial relationships with the target speech would tax speech perception. However, there was no significant interaction between Assessment point and Group; that is, musical training was not associated with changes in SiN perception. Interactions that were predicted to show a musicians’ advantage for SRTs under various masker and spatial manipulations were also not significant (Group × Assessment point × Masker type; Group × Assessment point × Spatial location). No other study to date has compared effects of musical training on SRTs in children using different masker types and target-masker spatial combinations in 5- to 7-year-old children. Therefore, in what follows, findings from previous cross-sectional and longitudinal studies which show parallels with the present study but were conducted with children of various ages as well adults will be considered.

In a cross-sectional study by Strait et al. (2012), 7- to 13-year-old children with at least 4 years of musical training or no musical training were tested on different SiN perception tasks. Consistent with the present study’s observations, the authors found no evidence for a musicians’ advantage for speech perception in collocated babble or SSN. However, there was an advantage for musicians’ speech perception when the SSN was spatially separated from the target speech. The masker and spatial conditions used in both studies had the potential to indicate whether musical training improves either peripheral auditory processing, cognition, or both. If the benefits of musical training were for peripheral auditory processing, speech perception under separated and energetic masker conditions would have been predicted because these conditions rely more on peripheral auditory processing than cognition. If benefits of musical training were cognitive, however, speech perception under the more cognitively demanding collocated and informational masker conditions would have been predicted in the musical-training group. In the case that both these processes were improved through musical training, both spatial and masker conditions would have shown improvement. As the cumulative findings of Strait et al. (2012) and the present study indicate no musicians’ advantage for collocated conditions accompanied by informational maskers (i.e., babble noise or a single talker, respectively), a cognitive advantage of musical training cannot be concluded. Although Strait et al. (2012) found a musicians’ advantage for speech perception under spatially separated energetic masker (i.e., SSN) conditions, the present study failed to demonstrate such trends longitudinally. Therefore, a benefit for musical training for peripheral auditory processing remains to be conclusively established.

A longitudinal musical-training study with children aged 6–9 years conducted by Slater et al. (2015) investigated whether musical training of up to 4 h per week over 2 years improves speech perception in collocated SSN compared to controls who received no musical training. After 1 year, the two groups did not perform significantly differently but a musicians’ advantage was found after the second year of training. The discrepancy between this observation and the present study’s findings might result from the considerable difference in the amount of the musical training provided in the two studies. However, cross-sectional studies with at least 4 years of musical training (Strait et al., 2012) and adults with over 10 years of musical training (Ruggles et al., 2014; Boebinger et al., 2015) reported no benefits for speech perception in collocated SSN for children either. Further longitudinal investigations are warranted to interpret these conflicting results.

### Effect of Musical Training on Phonological Short-Term Memory

A secondary aim of this study was to test if musical training improved phonological short-term memory, which, in turn, could mediate improvements in SiN perception. At baseline, the musical-training group showed significantly higher FDS scores and this advantage was maintained over time. Although groups were not equally matched at baseline, the ANOVA indicated whether the increase relative to baseline scores over time was greater in the musical training group than controls. The main effect of Assessment point indicated that FDS did not improve significantly over the course of 1 year across groups, and the non-significant interaction between Assessment point and Group meant that the relative increase in FDS was not higher in either group.

These findings contrast with results of Lee et al. (2007) who showed that 12-year-old children with an average of 6 years of musical training had better FDS than non-musicians, and results of Strait et al. (2012) who reported better auditory working memory in musically trained children aged 7 to 13 years. Strait et al. (2012) further reported that the correlation between the number of years of musical training received and auditory working memory ability was “marginally significant” (*r* = 0.38, *p* = 0.08), strongly implying that musical training was causally responsible for the measured between-group difference. Since the studies by Lee et al. (2007) and Strait et al. (2012) were cross-sectional, it cannot be excluded that these findings might be due to pre-existing between-group differences.

However, longitudinal evidence indicates that musically trained children’s phonological short-term memory advantage, indicated by cross-sectional studies, are not necessarily due to pre-existing differences masquerading as training effects. A study by Roden et al. (2014) showed that 45 min of weekly musical training over 1 year in 7- to 8-year-old children significantly improved phonological short-term memory capacity. Somewhat surprisingly, the present study, even though methodological very similar (using also a longitudinal design, a comparable cognitive test, similarly aged participants, and a musical-training regimen of similar duration and frequency) failed to find evidence for a musical training-based cognitive improvement.

### Correlations Between Speech-in-Noise Perception and Phonological Short-Term Memory

The strength of the relationships between phonological short-term memory and SiN perception was assessed using Pearson correlations between FDS scores and SRTs in the different masker and spatial conditions. Across groups, there was a significant moderate inverse correlation at T1 and T2 when the masker was collocated SSN. Similarly, Strait et al. (2012) found that auditory working memory correlated significantly with SiN perception in spatially separate SSN. Although spatial conditions differed, both studies found that the energetic masker used (i.e., SSN) covaried significantly with memory processes. This suggests that these cognitive skills are most useful when dealing with speech-perception challenges to the auditory periphery. However, it would be more intuitive to expect that cognitive skills should be useful when dealing with the more cognitively demanding maskers (i.e., informational maskers) and spatial conditions (i.e., collocated). Although, less obviously cognitively taxing conditions (e.g., spatially separated SSN maskers) could have a cognitive component for which stronger cognitive abilities could potentially provide benefits.

### Limitations

Most prior studies investigating the musicians’ advantage used a cross-sectional design, probably due to logistical and practical difficulties associated with the implementation of an actual musical-training intervention. For the present study, a longitudinal design was adopted so as to investigate possible causal relationships between the studied variables. To mimic a realistic context for a training program targeting typically developing young children, and also for logistic reasons, the musical training was delivered as part of the school curriculum. These choices imposed certain limits on the experimental design of the current study. First, the children were not randomly assigned to one of the two groups, limiting the causal claims that could be made by the present study. Their choice to attend the music-focused or sports-focused school determined their group membership. Hence, a bias in terms of participant characteristics (e.g., motivation, cognitive abilities) cannot be ruled out, even though all participants were normally performing pupils and the two groups did not differ in age or maternal socio-economic status. Second, the nature, amount and frequency of musical training was fixed by the curriculum in the music-focused school. It could be argued that other forms of or more musical training could have produced improvements in SiN perception and/or in phonological short-term memory capacity. However, it should be noted that studies using even less musical training have reported significant effects of musical training on cognitive abilities, such as improvements in phonological short-term memory after 45-min-long weekly training over 1 year (Roden et al., 2014) or in reading ability after 30-min-long weekly training for 8 months (Myant et al., 2008). Finally, although the present study considered some potential confounds (i.e., socio-economic status, hearing and language ability) that might have motivated children to take up musical training and might have led to pre-existing between-group inequalities, personality is an additional factor which has shown to be predictive of involvement in musical activities in adults and children (Corrigall et al., 2013; Swaminathan and Schellenberg, 2018). As personality was not measured, it was beyond the scope of this study to evaluate the extent to which this factor contributed to children’s motivations to attend the respective schools, and thus represents a potential confound that should be controlled for in future studies.

## Conclusion

This study assessed the impact of 1 year of musical instrument training on phonological short-term memory and SiN perception in children aged 5–7 years. Musical training improved neither phonological short-term memory, nor SiN perception in any of the listening conditions combining different maskers and spatial target-masker configurations that aimed to simulate realistic classroom conditions. This contrasts with previous studies in similarly aged children reporting evidence of musical-training benefits for SiN perception (Slater et al., 2015) and phonological short-term memory (Roden et al., 2014). While our study adds to the list of investigations failing to find evidence for a musicians’ advantage, more (especially longitudinal) research is warranted to investigate the nature, amount and frequency of musical training required for potential benefits in SiN perception and its underlying cognitive processes.

## Data Availability Statement

The datasets generated for this study are available on request to the corresponding author.

## Ethics Statement

The studies involving human participants were reviewed and approved by Faculty of Humanities Research Ethics Committee, University of Pretoria (GW20171130HS). Written informed consent to participate in this study was provided by the participants’ legal guardian/next of kin.

## Author Contributions

DM designed the study, collected and analyzed the data, wrote the manuscript, and prepared the tables. CF assisted with revising the manuscript and responding to reviewers comments. RE collected the data and provided comments. JL and RL supervised the project and provided comments.

## Conflict of Interest

The authors declare that the research was conducted in the absence of any commercial or financial relationships that could be construed as a potential conflict of interest.

## Footnotes

1 The musical training taught the following musical concepts: pitch (identify and produce high and low pitches, identify and produce pitch contours), duration (identify and produce long and short sounds), beat (keeping steady beat to music through movement and instrumental play), timbre (identify sounds through aural cues, identify instrument families), dynamics (getting louder and softer), form structure (introducing common form structures including AB, ABA, and Rondo form), rhythm (producing crotchet and quaver rhythmic patterns, creating own rhythmic patterns) and creativity (creating a “sound story”).
